# Pattern of anxiety, insecurity, fear, panic and/or phobia observed by quantitative electroencephalography (QEEG)

**DOI:** 10.1590/1980-57642018dn12-030007

**Published:** 2018

**Authors:** Valdenilson Ribeiro Ribas, Renata Guerra Ribas, Jean de Almeida Nóbrega, Marcília Vieira da Nóbrega, Juliana Azevedo de Andrade Espécie, Murilo Tolêdo Calafange, Clenes de Oliveira Mendes Calafange, Hugo André de Lima Martins

**Affiliations:** 1 Universidade Federal de Pernambuco Ringgold Standard Institution - Pós-Graduação em Neuropsiquiatria e Ciências do Comportamento, Recife, PE, Brazil.; 2 Cérebro e Tecnologia Neurofeedback Recife (CTNR) - Cursos/Pesquisas, Jaboatão dos Guararapes, PE, Brazil.; 3 Universidade Federal de Campina Grande Ringgold Standard Institution - Electronic Engineering, Campina Grande, PB, Brazil.; 4 Universidade Federal de Pernambuco Ringgold Standard Institution - Psychology, Recife, PE, Brazil.

**Keywords:** anxiety, fear, panic, phobia, quantitative electroencephalography (QEEG), ansiedade, medo, pânico, fobia, eletroencefalografia quantitativa (QEEG)

## Abstract

Stress is a response in which an individual wants to have more control over a situation. A constant state of stress is called anxiety. Some patients deny symptoms. An instrument can help arrive at a diagnosis.

**Objective:**

Using TQ-7 QEEG, this study aimed to evaluate the association of symptoms of anxiety, insecurity, fear, panic and phobia with hot temporals defined as Beta (15-23 Hz) >17% and High-Beta waves (23-38 Hz) >10% at T3 and T4.

**Methods:**

Five hundred and forty-three patients of both genders with ages ranging from 16-59 years were evaluated, divided into two groups: Control (without hot temporals: n=274) and Case Group (with hot temporals: n=269). The Chi-square test was used (p-values ≤0.05).

**Results:**

There was a significant association (p-value <0.001) between the symptoms related to amygdala activation, expressed in the temporals (Beta >17% and High-Beta >10%). (Anxiety, T3=89.6% - T4=88.8%; T3=92.6% - T4=93.3%), (Fear, T3=80.7% - T4=84.4%; T3=82.9% - T4=95.9%), (Insecurity, T3=82.2% - T4=81.4%; T3=69.5% - T4=97.8%), (Panic, T3=52.4 - T4=72.5%; T3=90.3% - T4=74.0%), (Phobia, T3=17.5% - T4=22.7%; T3=19.7% - T4=27.1%), when compared to the respective controls (Beta control, T3=8.4%, 10.2%, 21.2%, 1.1%, 0.4% and T4=11.3%, 4.4%, 23.0%, 2.6%, 1.1%) (High-Beta control, T3=4.0%, 6.9%, 6.2%, 0.4%, 0.0% and T4=17.5%, 6.2%, 3.3%, 4.0%, 0.7%).

**Conclusion:**

Anxiety, insecurity, fear, panic and phobia are observed by QEEG when the levels of total Beta >17% and High-Beta waves >10% at T3 and T4.

## Resumo

O estresse é uma resposta a uma situação na qual, um indivíduo quer ter mais controle. Um estado de estresse constante é chamado de ansiedade. Alguns pacientes negam sintomas. Um instrumento pode auxiliar um diagnóstico.

**Objetivo:**

Utilizando o QEEG TQ-7, este estudo objetivou avaliar a associação dos sintomas de ansiedade, insegurança, medo, pânico e/ou fobia com a categoria de temporais quentes definidos como Beta (15-23 Hz) >17% e Beta-Alta (23-38 Hz) >10% em T3 e T4.

**Métodos:**

Foram avaliados 543 pacientes de ambos os gêneros na faixa etária de 16-59 anos, divididos: Controle (sem temporais quentes: n=274) Grupo estudado (com temporais quentes: n=269). Foi utilizado o teste qui-quadrado (p-valor ≤0.05).

**Resultados:**

Houve associação significativa (p-valor <0.001) entre os sintomas relacionados à ativação da amigdala, expressos nos temporais (Beta >17% e Beta-Alta >10%). (Ansiedade, T3=89,6% - T4=88,8%; T3=92,6% - T4=93,3%), (Medo, T3=80,7% - T4=84,4%; T3=82,9% - T4=95,9%), (Insegurança, T3=82,2% - T4=81,4%; T3=69,5% - T4=97,8%), (Pânico, T3=52.4% - T4=72.5%; T3=90.3% - T4=74.0%), (Fobia, T3=17,5% - T4=22,7%; T3=19,7% - T4=27,1%), quando comparados aos respectivos controles (Beta controle, T3=8,4%, 10,2%, 21,2%, 1,1%, 0,4% e T4=11,3%, 4,4%, 23,0%, 2,6%, 1,1%) e (Beta-Alta Controle, T3=4,0%, 6,9%, 6,2%, 0,4%, 0,0%; T4=17,5%, 6,2%, 3,3%, 4,0%, 0,7%).

**Conclusão:**

Ansiedade, insegurança, medo, pânico e fobia são observados no QEEG, quando Beta >17% e Beta-Alta >10% em T3 e T4.

Anxiety is an emotional state with physiological^1^ or psychological components.^2^ As part of the normal spectrum of human experiences, it is a driving force behind human performance.^3^ However, it becomes pathological when it is dysfunctional in proportion to the situation that triggers it, or when there is no reason for its existence.^4^

Some factors have been listed as having relevant roles in the etiology of pathological anxiety. These include dissatisfaction at work,^5^ a feeling of restlessness and stressful environments at home or work^6^ and, above all, repetitive thoughts such as the habit of complaining (in the sense of lamentation),^7^ over-analyzing, criticizing excessively, continuously judging and always thinking about the future and the past.^8^^,^ 9

Hence, it seems important to understand that pathological anxiety is a multifactorial disease,^10^ because there are psychological and/or neurophysiological components. Psychological components include low control by psychic defenses (rationalization, reactive formation, sublimation, projection, regression, transference and displacement),^11^^,^ 12 which can be measured using the Rorschach Projective test.^13^^,^ 14 Neurophysiological components, on the other hand, include low functional reserves (vitamins, proteins, amino acids and lipids) at the level of neuroglia (astrocytes, oligodendrocytes and/or Schwann cells)^15^ causing a low baroreflex rate,^16^ alterations in the physiological response observed by peripheral temperature^17^ and adrenal exhaustion.^18^

Thus, when one thinks of measuring anxiety from the physiological point of view, some professionals do so by adrenal response controlled by the autonomic nervous system (sympathetic and parasympathetic). The central nervous system has received prominence only in the neurochemical context, that is, in relation to neurotransmitters.

Hence, this study aimed to demonstrate that there is an expected pattern that identifies anxiety through brain electrical activity using quantitative electroencephalography (QEEG), which can help physicians and other healthcare professionals diagnose subjects and, consequently, to choose the best treatment.

## METHODS

### Subjects

Five hundred and forty-three patients of both genders aged between 16 and 59 were evaluated at the Recife Brain and Neurofeedback Clinic (CTNR) under standard conditions at a temperature of 20±2ºC. In this cross-sectional, experimental, exploratory study, all the evaluations were performed in a single exam using QEEG of the TQ-7 neurofeedback system. The inclusion criterion was confirmation of a clinical diagnosis of anxiety by a physician, according to the Diagnostic and Statistical Manual of Mental Disorders (DSM V) criteria:

- Excessive anxiety and worry, occurring on most days over more than six months and linked to events and activities (e.g. work and school performance);
- Worry is difficult to control;
- Anxiety and worry are associated with three (or more) of the following symptoms (with at least some symptoms being present on most days in the last six months): restlessness or a feeling of being on edge, getting tired easily, difficulty concentrating, irritability, muscle tension, and sleep disturbances (difficulty falling or staying asleep and having unsatisfying sleep);
- Physical symptoms, worry or anxiety cause clinically significant distress or impairment in social, occupational, or other activities;
- The disturbance cannot be attributed to a general medical condition, substance use or other mental disorder.

Patients under 16 years old were not included in this study.

### Characterization of the TQ-7 method

The TQ-7 is an evaluation method called Trainers’ QEEG, which is in version 7,^19^ a component of The Learning Curve (TLC) technique, based on a protocol of 6 categories (disconnected, hot temporal lobes, reversals or asymmetry, blocking, locking, filtering and processing). It is described in the study: “The Learning Curve in Neurofeedback of Peter Van Deusen: a Review Article,” published in this journal, Dementia & Neuropsychology in 2016.^19^^,^ 20

The TLC Assessment or Trainers’ QEEG (TQ) method^19^ is enabled through the Q-wiz amplifier and the Bioexplorer program.^20^^,^ 21 The Q-wiz has four simultaneous 24-bit channels and a maximum sampling rate of 512 samples per second; each channel has a high pass filter of 0.2 Hz and the signals are analyzed by a program called Bioexplorer. Bioexplorer was developed by CyberEvolution Inc. for real time biological signal acquisition, processing, display, recording and playback. It permits the user to create a setup (or ‘design’) graphically in order to process the raw signals from the Q-wiz amplifier by interconnecting different processing, display and audio objects for biofeedback.^21^ The processing objects include several types of low-pass, band-pass and high-pass filters, fast Fourier transforms (FFTs), and mathematical and logical operations. The audio and display objects include display bars with automatic and manual thresholds, graphical trends, power spectrum, audio and MIDI playback, video player and flash game players, all of which are controlled by processing objects.^21^

**Figure 1:**
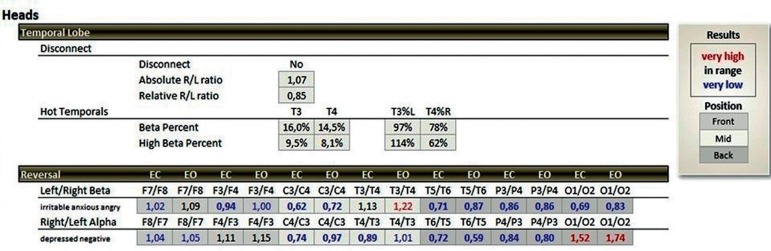
Percentage expected quantity of beta waves (15-23 Hz) and Beta-Alta (23-38 Hz) at the T3 and T4 points of the 10-20 electroencephalography system in a summary statistical distribution (TQ-7 analyze page), expressed in black color, representing the control group.

**Figure 2:**
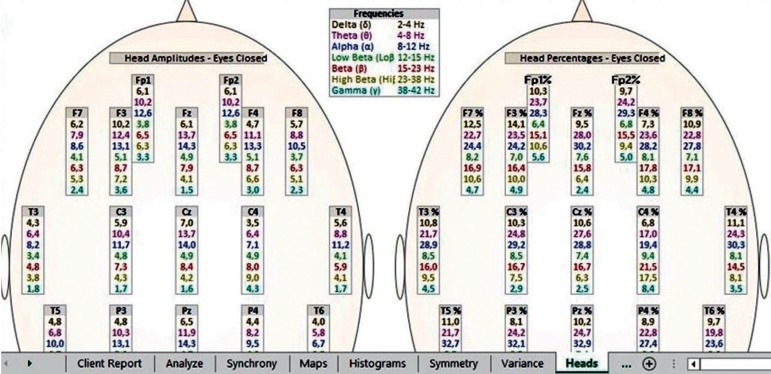
The expected percentage of Beta-wave (15-23 Hz) and Beta-High (23-38 Hz) waves at the T3 and T4 points of the 10-20 electroencephalography system in a broad statistical distribution (TQ-7 heads page), expressed in black color, representing the control group.

**Figure 3:**
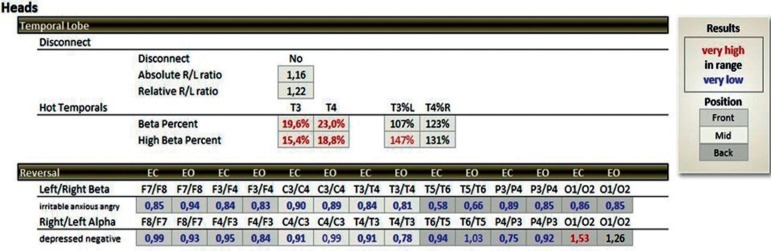
Percentage quantity above the expected beta wave (15-23 Hz >17%) and Beta-High (23-38 Hz >10%) at the T3 and T4 points of the 10-20 electroencephalography system in a summary statistical distribution (TQ-7 analyze page), expressed in red color, representing the group studied (hot temporal lobes).

**Figure 4:**
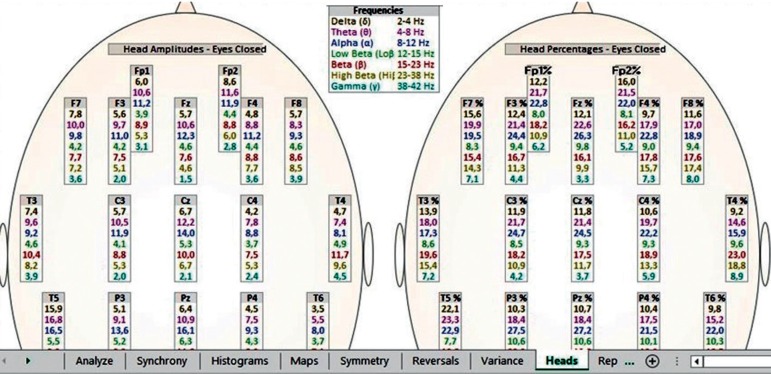
Percentage quantity above the expected beta wave (15-23 Hz >17%) and Beta-High (23-38 Hz >10%) at the T3 and T4 points of the 10-20 electroencephalography system in a broad statistical distribution (TQ-7 heads page), expressed in red color, representing the group studied (hot temporal lobes).

The patterns investigated are related to the activation of T3 and T4 temporal electrodes of the 10-20 EEG system, where values between 14 and 17% of beta waves (15-23 Hz) and a maximum of 10% of high-beta waves (23-38 Hz) are expected^20^ (Figure 1 and Figure 2). When values above these percentages are observed, the condition is called hot temporal lobe according to the investigative standard of The Learning Curve (TLC) in neurofeedback by Peter Van Deusen^19^^,^ 20 (Figure 3 and Figure 4). In these cases, one would expect to find symptoms of anxiety, fear, insecurity, panic and/or phobia.^20^ During the evaluation using the TQ-7 method, the electrodes are placed according to the rules of the 10-20 system.^22^ The evaluation is performed with eyes closed, eyes open, and open performing a task.^23^ The task of each standard site is designed to activate the site and is measured using an algorithm that calculates the total percentage of each frequency band of brainwaves.^23^ To assess the C3, C4, T3 and T4 sites, the evaluator tells a story for the patient to remember the details; F3, F4, P3 and P4 are activated when the respondent is asked to remember several sequences of numbers; the F7, F8, T5 and T6 sites are activated by interpreting a text; the Fz, Pz, Cz and Oz sites are activated when the patient thinks about their plans for the following day; and the Fp1, Fp2, O1 and O2 sites are evaluated by counting the number of times the letter ‘a’ appears in a short text.^19^^,^ 21

A maximum of 30% of beta (15-23 Hz) and high-beta (23-38 Hz) waves is still expected at all major points of the 10-20 EEG system, compared to the sum of the percentages of the average and slow waves, especially in the posterior area of the brain, specifically at P3, P4, T5, T6, O1 and O2.^20^^,^ 23 Values above 30% indicate a type of anxiety linked to issues of worry and indicate a pattern of brain electrical activity related to difficulty reducing activation for nocturnal rest.^23^

### The groups

This cross-sectional study was performed in subjects with ages ranging from 16 to 59 years divided into two groups: the Control Group was made up of male and female patients without hot temporal lobes (n=274) and the Case Group comprised patients of both genders with hot temporal lobes (n=269).

We used the following equation to calculate the sample size for an infinite population:

$$n=\frac{z^{2}*P*Q}{e^{2}}$$

Where

*n* = sample size;

*z* = 1.96 (according to table of area under normal curve for the given 95% confidence level);

*P* = 0.5 = 50% to succeed;

*Q* = 0.5 = 50% of failure;

*e* = estimate of the percentage of the true value;

e^2^= 5% (for an expected estimate of 5% of the true value).

### Data analysis

The data were analyzed using the Chi-square test with significance set for p-values ≤0.05. The results are expressed as frequencies and percentages and represented in a contingency table.

## RESULTS

There was a significant association (p-value <0.001) between the symptoms related to amygdala activation expressed in the temporal lobe, and the increase in beta waves (15-23 Hz) >17% and temporal lobe symptoms T3 = Anxiety (241/269, 89.6%), Fear (217/269, 80.7%), Insecurity (221/269, 82.2%), Panic (141/169, 52.4%), Phobia (47/169, 17.5%) (Table 1) and T4 = Anxiety (239/269, 88.8%), Fear (227/269, 84.4%), Insecurity (219/269, 81.4%), Panic (195/269, 72.5%), Phobia (61/269, 22.7%) (Table 3), when compared to the respective controls (T3=8.4%, 10.2%, 21.2%, 1.1%, 0.4% (Table 1) and T4=11.3%. 4.4%, 23.0%, 2.6%, 1.1%) (Table 3).

**Table 1:** Evaluation of symptoms according to group: Percent of Beta (15-23 Hz) wave in left temporal lobe (T3).

Variables		Group				p-value
		Control (Less than 17%)		Hot temporal lobes (More than 17%)		
		n	%	N	%	
Total		274	100.0	269	100.0	
Anxiety	Yes	23	8.4	241	89.6	p** <0.001*
	No	251	91.6	28	10.4	
Fear	Yes	28	10.2	217	80.7	p** <0.001*
	No	246	89.8	52	19.3	
Insecurity	Yes	58	21.2	221	82.2	p** <0.001*
	No	216	78.8	48	17.8	
Panic	Yes	3	1.1	141	52.4	p** <0.001*
	No	271	98.9	128	47.6	
Phobia	Yes	1	0.4	47	17.5	p** <0.001*
	No	273	99.6	222	82.5	

* Significant difference at 5.0%.

** Pearson Chi-square test.

**Table 2:** Evaluation of symptoms according to group: Percent of High Beta (23-38 Hz) wave in left temporal lobe (T3).

Variables		Group				p-value
		Control (Less than 10%)		Hot temporal lobes (More than 10%)		
		n	%	N	%	
Total		274	100.0	269	100.0	
Anxiety	Yes	11	4.0	249	92.6	p** <0.001*
	No	263	96.0	20	7.4	
Fear	Yes	19	6.9	223	82.9	p** <0.001*
	No	255	93.1	46	17.1	
Insecurity	Yes	17	6.2	187	69.5	p** <0.001*
	No	257	93.8	82	30.5	
Panic	Yes	1	0.4	243	90.3	p** <0.001*
	No	273	99.6	26	9.7	
Phobia	Yes	0	0.0	53	19.7	p** <0.001*
	No	274	100.0	216	80.3	

* Significant difference at 5.0%.

** Pearson Chi-square test.

**Table 3:** Evaluation of symptoms according to group: Percent of Beta (15-23 Hz) wave in left temporal lobe (T4).

Variables		Group				p-value
		Control (Less than 17%)		Hot temporal lobes (More than 17%)		
		n	%	N	%	
Total		274	100.0	269	100.0	
Anxiety	Yes	31	11.3	239	88.8	p** <0.001*
	No	243	88.7	30	11.2	
Fear	Yes	12	4.4	227	84.4	p** <0.001*
	No	262	95.6	42	15.6	
Insecurity	Yes	63	23.0	219	81.4	p** <0.001*
	No	211	77.0	50	18.6	
Panic	Yes	7	2.6	195	72.5	p** <0.001*
	No	267	97.4	74	27.5	
Phobia	Yes	3	1.1	61	22.7	p** <0.001*
	No	271	98.9	208	77.3	

* Significant difference at 5.0%.

** Pearson Chi-square test.

There was a significant association (p-value <0.001) between the symptoms related to amygdala activation expressed in the temporal lobe and the increase in high-beta waves (23-38 Hz) and temporal lobe symptoms T3: Anxiety (249/269: 92.6%), Fear (223/269: 82.9%), Insecurity (187/269: 69.5%), Panic (243/269: 90.3%) and Phobia (53/269: 19.7%) (Table 2) and T4: Anxiety (251/269: 93.3%), Fear (258/269: 95.9%), Insecurity (263/269: 97.8%), Panic (199/269: 74.0%) and Phobia (73/269: 27.1%) (Table 4), when compared to the respective controls (T3=4.0%, 6.9%, 6.2%, 0.4%, 0.0% (Table 2); T4=17.5%, 6.2%, 3.3%, 4.0%, 0.7%) (Table 4).

**Table 4:** Evaluation of symptoms according to group: Percent of High Beta (23-38 Hz) wave in left temporal lobe (T4).

Variables		Group				p-value
		Control (Less than 10%)		Hot temporal lobes (More than 10%)		
		n	%	N	%	
Total		274	100.0	269	100.0	
Anxiety	Yes	48	17.5	251	93.3	p** <0.001*
	No	226	82.5	18	6.7	
Fear	Yes	17	6.2	258	95.9	p** <0.001*
	No	257	93.8	11	4.1	
Insecurity	Yes	9	3.3	263	97.8	p** <0.001*
	No	265	96.7	6	2.2	
Panic	Yes	11	4.0	199	74.0	p** <0.001*
	No	263	96.0	70	26.0	
Phobia	Yes	2	0.7	73	27.1	p** <0.001*
	No	272	99.3	196	72.9	

* Significant difference at 5.0%.

** Pearson Chi-square test.

## DISCUSSION

This study found an association between the symptoms of anxiety, fear, insecurity, panic and phobia, and the category of hot temporal lobes (T3 and T4) of the TLC technique using the TQ-7 method.

Although, these findings corroborate data gathered in the experiments of Van Deusen (1994-2001) *apud* Ribas et al. (2016),^20^ not all patients present these five symptoms simultaneously; there are patients exhibiting from four to only one of the symptoms described above. This phenomenon, which allows differentiation between the perceptions of the symptoms, seems to be related to the difference between the levels of functioning of psychic defense mechanisms that allow lower levels of anxiety and stress, even with the support of auxiliary egos or placebos of the masses, such as superstitions, rituals and faith expressed in religious spaces.^24^

Thus, it seems relevant to make the following analogy: just as the DSM-5 considers the etiology of multifactorial mood disorder (psychological, environmental, biological and/or genetic),^25^ one can imagine that anxiety control can also be achieved via multifactorial forms (psychological, amygdalae, adrenal and cognitive concentration). The example mentioned in the previous paragraph, about psychic defense mechanisms, can explain the control of anxiety psychologically.^11^^,^ 26

The mechanisms of psychic defenses referred to in the previous paragraph, emerged as follows: the neurologist Sigmund Freud observed that his patients presented ways of thinking and acting that reduced their levels of anxiety and stress, which he generically called psychic defense mechanisms. He then classified them as rationalization, reactive formation, sublimation, fixation, repression, regression, projection, denial, and transfer, among others.^27^

However, if individuals do not have a sound family structure that allows them to acquire these defenses to protect them from the persistent discomforts of anxiety, the neurochemical and electrical functioning of the autonomic and central nervous systems is eventually affected.

The human physiological response to stress was fully adapted to protect people from danger^28^ during the life of our ancestors when they had to flee from dangerous animals. It is a fight or flight reaction,^29^ when the body needs strength to protect itself from danger. The activation of the temporal lobes, specifically T3 and T4, seems to indicate an excessive activation of the amygdalae.^30^

The amygdalae act as a sensor of threats or a lack of control, communicating the need for a reaction to the hypothalamus. The hypothalamus, in turn, releases corticotropin-releasing hormone (CRH), which binds to the adenohypophysis that then produces the adrenocorticotrophic hormone (ACTH). ACTH binds to the adrenal cortex and adrenal medulla.^31^

Beta waves begin to appear in humans when they are between 5 and 6 years of age and have generators in different locations.^32^ Low-beta waves (12-15 Hz) are generated in the thalamic nuclei and cortex, especially in the sensorimotor area,^33^ whereas beta waves (15-23 Hz) and high-beta waves (23-38 Hz) are generated in the cortex.^34^ Low-beta and beta waves indicate that the individual is conscious and processing, whereas high-beta waves are responsible for alerting the individual through fear, anxiety and hypervigilance.^35^ The response to anxiety through the amygdala can be observed by excess beta waves (15-23 Hz) above 17% and by excess high-beta waves (23-38 Hz) above 10% throughout the brain, specifically, in temporal lobes (T3 and T4).^20^

To understand the control of adrenal anxiety, it seems relevant to understand its anatomical and functional formation. The adrenal cortex is divided into three parts. The glomerulosa produces mineralocorticoids, a class of steroid hormones, with aldosterone being the main hormone of this class. Aldosterone has the function of controlling the metabolism of water and sodium in blood plasma.^36^ The fasciculata produces glucocorticoids, with cortisol being the most important.^37^ Thirdly, the reticularis (innermost region) produces sex hormones, such as estrogen, progesterone, testosterone and dehydroepiandrosterone (DHEA).^38^^,^ 39

The inner region, the adrenal medulla, produces catecholamines (Dopamine, Noradrenaline and Adrenaline). The production of catecholamines begins with the action of the enzyme tyrosine hydroxylase, a cytosolic enzyme only found in cells that contain catecholamines, which adds a hydroxyl to the compound tyrosine to form dihydroxyphenylalanine (DOPA). This first step of hydroxylation is the main control point in the synthesis of noradrenaline. Next, norepinephrine is converted into adrenaline by the phenylethanolamine-N-methyl transferase enzyme in the adrenal gland.^40^^,^ 41

Adrenaline is an important hormone in the response to stress because it increases glucagon concentration, which stimulates an increase in glucose and blocks insulin production thereby increasing heart rate, decreasing gastrointestinal motility, causing mydriasis, and increasing muscle energy. Thus, the adrenal gland controls the activation of the state of alert. However, if it remains activated or if it works constantly with a low functional reserve (amino acids, proteins, vitamins, minerals, lipids and complex carbohydrates), it can evolve to exhaustion, impairing the control of anxiety.^18^

Thus, even though this study found an association between anxiety, fear, insecurity, panic and phobia with high activation of the amygdalae expressed in the T3 and T4 regions, it is recognized that it is not the only region that can measure or control anxiety.

Another way to identify an anxious brain that was not investigated in this study but may be the subject of future studies is the excessive activation of the posterior region of the brain, indicating that the subject is thinking a lot about the past, analyzing, criticizing or judging. This is because most blood flow will certainly be in the posterior region of the brain as blood enables the firing of active neurons after the second (Krebs cycle) and the third stage of cellular respiration (oxidative phosphorylation) with conversion of energy sources: carbohydrates, proteins and lipids into adenosine triphosphate and exothermic energy.^42^

Hence, when this blood flow is concentrated at the back of the brain, it generates anxiety because the subject is not doing anything, the present moment is not lived, nothing can be done to change events because the subject continuously thinks of the past and the future. In this sense, it is observed that concentration is another form of anxiety control.^43^^,^ 44

These findings are very relevant because they may allow physicians to use the QEEG examination to complement their clinical diagnosis of anxiety. This is especially true in those patients who are not very self-aware or who have difficulty reporting their complaints, making them more susceptible to heart attack or stroke due to their high levels of untreated anxiety.
